# Sleeve Gastrectomy Is Associated with a Greater Reduction in Plasma Liver Enzymes Than Bypass Surgeries—A Registry-Based Two-Year Follow-Up Analysis

**DOI:** 10.3390/jcm10051144

**Published:** 2021-03-09

**Authors:** Shira Azulai, Ronit Grinbaum, Nahum Beglaibter, Shai Meron Eldar, Moshe Rubin, Rachel Ben-Haroush Schyr, Orly Romano-Zelekha, Danny Ben-Zvi

**Affiliations:** 1 Department of Developmental Biology and Cancer Research, Institute for Medical Research Israel-Canada, The Hebrew University of Jerusalem-Hadassah Medical School, Jerusalem 91120, Israel; shira.azulai@gmail.com (S.A.); Rachel.schyr@mail.huji.ac.il (R.B.-H.S.); 2 Department of Surgery, Hadassah-Hebrew University Medical Center, Mount Scopus, Jerusalem 91240, Israel; ronitgr@hadassah.org.il (R.G.); bnahum@hadassah.org.il (N.B.); 3 Bariatric Surgery Unit, Division of General Surgery, Tel Aviv Sourasky Medical Center, Tel-Aviv 64239, Israel; shaime@tlvmc.gov.il; 4 Israel Sackler Faculty of Medicine, Tel-Aviv University, Tel-Aviv 69978, Israel; moshe.rubin2@sheba.health.gov.il; 5 Department of Surgery, Tel HaShomer Medical Center, Ramat Gan 52621, Israel; 6 Israel Center for Disease Control, Ministry of Health, Ramat Gan 52621, Israel; orly.romano@moh.gov.il

**Keywords:** bariatric surgery, logistic model, ALT, weight-loss

## Abstract

Bariatric surgeries may lead to an improvement in metabolic fatty liver disease, and a reduction in the levels of the hepatic enzyme Alanine Aminotransferase (ALT). We compared the effects of Sleeve Gastrectomy (SG), Roux en Y Gastric Bypass (RYGB) and One Anastomosis Gastric Bypass (OAGB) on the levels of ALT by analysis of two-year follow-up data from 4980 patients in the Israeli Bariatric Registry that included laboratory tests and demographic information. Pre-operative characteristics of patients, and particularly levels of liver enzymes, were similar across surgery types. Regression modeling and retrospective matching showed that SG was superior to RYGB and OAGB in reducing ALT levels, and in reducing the fraction of patients with abnormally high ALT levels. Two-year post-surgery, an increase in ALT levels from normal to abnormal levels was observed in 5% of SG patients, and in 18% and 23% of RYGB and OAGB patients. In conclusion, SG leads to a greater reduction in ALT levels compared with bypass surgeries and a lower incidence of post-surgical elevation of ALT levels. Further studies are required to identify the cause for the rise in liver enzymes, and to determine whether ALT levels correlate with liver pathology especially following bariatric surgery.

## 1. Introduction

Bariatric surgery leads to a reduction in the degree of liver fibrosis, improves the severity of non-alcoholic steatohepatitis (NASH) and non-alcoholic fatty liver disease (NAFLD), and leads to a reduction in the plasma levels of the liver enzymes alanine aminotransferase (ALT) and aspartate aminotransferase (AST) in patients with obesity, in short and long-term studies [1,2,3,4,5,6,7,8,9]. Sustained weight-loss can also induce improvement in NASH and hepatic steatosis [10,11], and drastic weight-loss is considered one of the main forces driving improvement in hepatic disease following bariatric surgery.

While all bariatric surgery generally improves obesity, different types of surgery differ in resulting anatomy, potential mechanisms of action [12] and outcome: bypass surgery such as Roux en Y Gastric Bypass (RYGB) and One Anastomosis Gastric Bypass (OAGB) usually induce greater weight-loss and reduction in glycated hemoglobin (A1C) than Sleeve Gastrectomy (SG) [13,14,15,16] and are therefore expected to be superior in improving the outcomes of NAFLD. RYGB was shown to be superior to gastric banding, in long-term histological studies in improving NASH [3,8] and OAGB shows great promise in improving NASH in histological studies [17].

In this study, we analyzed data from 4980 patients in the Israeli Bariatric Registry [18,19,20] to compare how SG, RYGB and OAGB affect the plasma levels of ALT two years after surgery. ALT is a basic biomarker for liver injury and its blood levels increase as a result of several conditions, including NAFLD, alcoholic steatohepatitis and other hepatic diseases. According to the American College of Gastroenterologists, ALT values of over 25IU/L in women and 33 IU/L in men [21] are considered abnormally high. Other common ALT cutoff values of 40 or 50 IU/L do not consider sex [22,23,24]. There is no consensus on abnormal levels of AST [21]. A previous, one-year post-op study on the same registry [18] has surprisingly found that OAGB induces greater weight-loss, but a higher incidence of very high (>50 IU/L) ALT levels, than other bariatric surgeries. Given new studies showing the positive histological outcomes of bypass surgery on NASH, we hypothesized that RYGB and OAGB will reduce the fraction of patients with abnormal ALT levels when considering a longer follow-up and after accounting for the pre-operative differences between patients opting for OAGB, RYGB or SG.

## 2. Materials and Methods

### 2.1. Study Population

The study was approved by the review board of the Center for Disease Control of the Israeli Ministry of Health. We analyzed data in the Israeli Bariatric Surgery registry, from January 2014 to April 2016. Clinical parameters recorded were surgery type (SG, RYGB, OAGB, other) body mass index (BMI), lab tests for plasma ALT, AST, triglycerides and percentage of A1C, and the existence of hypertension and obstructive sleep apnea (OSA). Demographic parameters were ethnicity (Jewish, Arab, other), sex, age, alcohol consumption and smoking status. Alcohol consumption was self-reported and refers to more than occasional consumption. Smoking was self-reported and refers to regular daily smoking. Over 90% of the patients that underwent SG, RYGB or OAGB were either Jewish or Arab. Other ethnicities and surgery types were excluded from the analysis. Two-year post-surgery data on ALT were available on 4980 patients. This dataset was used in the analyses presented in this study (Supplementary Table S1). Abnormally high ALT levels were defined as greater than 33 IU/L for males and 25 IU/L for females [21].

### 2.2. Statistics

Continuous variables are reported as median with interquartile range in square brackets and categorical variables are reported as the number of patients with the percentage in round brackets. The χ^2^ test was applied for categorical variables. Mann-Whitney U-test or Kruskal-Wallis H-tests were used for continuous variables. We applied the Bonferroni correction method to account for multiple testing of parameters.

We performed 1:1:1 retrospective matching between SG, RYGB and OAGB patients. We demanded an exact match in categorical values (sex, ethnicity, hypertension comorbidity) and a minimal Euclidian distance in continuous parameters (age, A1C, BMI, ALT, triglycerides), with a predefined maximal distance.

Backward elimination multivariate logistic regression models were applied on variables that were statistically different between abnormally high and normal ALT levels two-years after surgery within the matching population. We used the Null hypothesis as a control model and used Log-Likelihood Ratio and Wald test to evaluate the model. The logistic model for high ALT levels two years after surgery included age, surgery type, sex, ethnicity, BMI, TG, A1C and hypertension. ALT was included in the model presented in Supplementary Table S2.

Scikit-learn, statsmodels, and SciPy packages were used to analyze the data [25,26,27].

## 3. Results

### 3.1. Study Population

The study population included 4980 patients within the Israeli Bariatric Registry that underwent primary bariatric surgery, for which pre-surgery and two-year follow-up data on ALT levels were available. Demographic data on sex, age and ethnicity and pre-operative BMI, ALT and A1C were available for these patients (Supplementary Table S1).

### 3.2. Sleeve Gastrectomy Is Associated with Lower ALT Levels Two Years after Surgery

**Figure 1:**
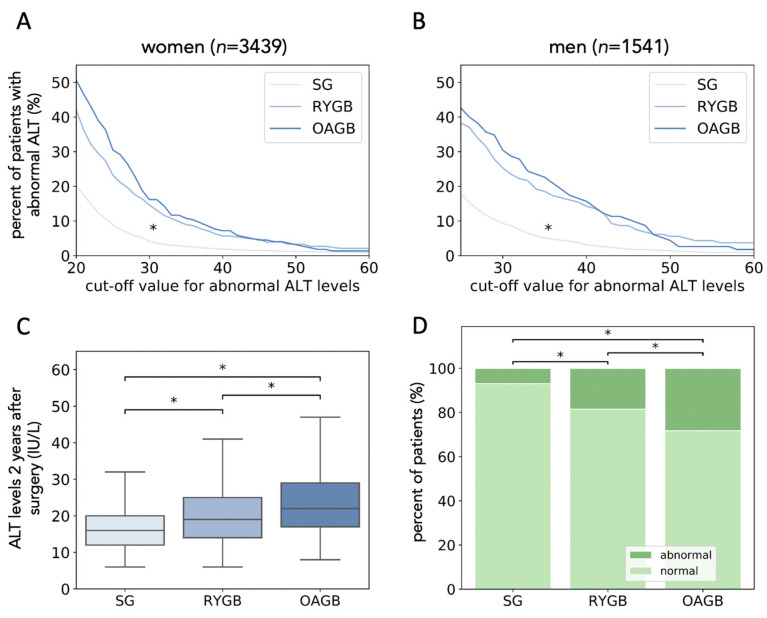
SG reduces the probability of abnormal ALT levels in the study population and retrospectively matched cohort. A-B: The percentage of female (**A**) and male (**B**) patients with abnormal ALT levels as a function of the cutoff defining abnormal ALT levels, per surgery. * *p* < 0.001 using Kruskal Wallis H test followed by Mann-Whitney U-test for pairwise comparison of SG with RYGB and SG with OAGB. (**C**): ALT levels two years after surgery in matched populations. *n* = 319 per surgery. * *p* < 0.01. (**D**): The percentage of patients with high ALT levels per surgery in matched populations. * *p* < 0.01 using the χ^2^ test. SG: Sleeve Gastrectomy; RYGB: Roux en Y Gastric Bypass; OAGB: One Anastomosis Gastric Bypass; ALT: Alanine Aminotransferase.

All surgery led to a reduction in the median levels of ALT and AST. We observed that patients who underwent SG had much lower levels of ALT and AST two years after surgery compared with RYGB and OAGB in both sexes, and the percentage of patients with abnormally high ALT levels [21] was lowest in SG (Table 1). Excess weight loss (EWL) was smallest in SG and highest in OAGB (Supplementary Table S3). There were no differences in pre-surgical ALT and AST levels and the fraction of patients with abnormal levels of ALT pre-surgery between the surgical groups. The fraction of patients with high ALT levels two years after surgery was largest in patients who had OAGB, slightly smaller in those who had RYGB and smallest in those who had SG for both sexes and a wide range of cutoff ALT levels (Figure 1A,B).

### 3.3. Retrospective Matching of Patients Shows That SG Is Superior in Reducing ALT Levels

Besides surgery type, patients with abnormal levels of ALT two years after surgery had different characteristics than those with normal ALT levels in terms of age, sex, ethnicity, and preoperative hypertension, A1C and ALT (Table 2). We retrospectively matched 957 patients who underwent SG, OAGB or RYGB (*n* = 319 per group) for age, sex, ethnicity, and pre-surgical hypertension, BMI, A1C, TG and ALT levels (Methods, Supplementary Table S4). Two-year follow-up data showed that patients who had SG displayed lower ALT levels than those who had RYGB or OAGB. Furthermore, the fraction of patients who had abnormally high ALT levels was smallest in the SG group of patients in both sexes and largest in the OAGB group: 28% of the patients had abnormal ALT levels following OAGB, 4 times more than after SG. (Figure 1C,D).

### 3.4. Surgery Type Has a Strong Effect on the Occurrence of High ALT Levels Two Years after Surgery

We constructed a backward elimination multivariate logistic model on the matched population, to test which pre-surgical factors are associated with clinically abnormal ALT levels two years after surgery [21]. We included parameters that had a significant difference (*p*-value ≤ 0.1) between patients with normal and abnormal ALT levels two years post-surgery (Table 2) and excluded pre-operative ALT levels. Within this model, surgery type had a strong effect with RYGB and OAGB increasing the odds ratio of having abnormal ALT levels by 3.05 and 5.40-fold respectively compared with SG (Table 3). Older age also increased the chances of having abnormal ALT levels two years after surgery. Female sex, Jewish ethnicity, pre-surgical A1C, BMI, TG and hypertension had no significant contribution and were eliminated from the model. A model which includes the entire dataset and initial levels of ALT shows similar results for surgery type. Furthermore, high pre-surgical levels of ALT increased the chances of having abnormal ALT levels two years after surgery (Supplementary Table S2).

### 3.5. Sleeve Gastrectomy Is Superior to Bypass Surgery in Normalizing ALT Levels

**Figure 2:**
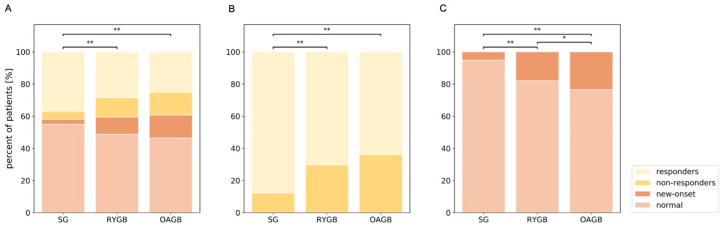
Sleeve gastrectomy has a higher probability for normalizing ALT levels and preventing new onset of abnormal ALT levels. (**A**) The percentage of patients who started with high levels of ALT and experienced a reduction to normal ALT levels (responders), started and remained with abnormal levels of ALT (non-responders), started with normal ALT levels that increased to abnormal levels (new-onset) or started and remained within the normal range (normal). Dark shades denote patients with abnormal ALT levels two years after surgery. *n* = 4144 SG; 498 RYGB; 338 OAGB. (**B**) Percentage of patients who started with abnormal levels of ALT and experienced a reduction to normal range (responders) or remained at abnormal ALT levels two years after surgery (non-responders). *n* = 1741 SG; 202 RYGB; 133 OAGB. (**C**) Percentage of patients whose ALT levels increased from normal to abnormal levels (new-onset), or started with normal levels of ALT and remained at normal range (normal). *n* = 2403 SG; 296 RYGB; 205 OAGB. ** *p* < 0.001, * *p* < 0.05 using χ^2^ test. SG: Sleeve Gastrectomy; RYGB: Roux en Y Gastric Bypass; OAGB: One Anastomosis Gastric Bypass; ALT: Alanine Aminotransferase.

We divided patients into four groups: those who had normal ALT levels before and two-years after surgery (normal), those whose levels normalized after surgery (responders), those whose ALT levels were high before and after surgery (non-responders) and those who had normal ALT levels before surgery, but high ALT levels two years after surgery (new-onset). There was a significant difference in the distribution of patients in each category according to surgery type (Figure 2A). In particular, 88% of patients with pre-surgical high ALT levels who had SG were responders, while only 70% of RYGB and 64% OAGB patients normalized their ALT levels (Figure 2B). Notably, responders and non-responders had similar pre-surgical ALT, lost similar weight in the two years after surgery, and ended up with similar A1C per surgical group (Supplementary Table S5).

### 3.6. Bypass Surgery Is Associated with New-Onset of High ALT Levels Despite Weight-Loss

New onset of high ALT levels was rare in patients who underwent SG (5%). The levels of ALT increased from normal to abnormal in 18% of the patients who had RYGB and 23% of those that had OAGB (Figure 2C). This increase occurred despite significant weight-loss (SG: 13.55 (9.97–17.16) kg/m^2^, RYGB: 14.54 (12.08–17.72) kg/m^2^ and OAGB: 15.00 (11.35–19.49) kg/m^2^). Pre-surgical ALT levels were not different between the groups. These results indicate that bypass surgery can cause an increase in ALT levels in a large fraction of patients.

## 4. Discussion

We analyzed data from thousands of patients in the Israeli Bariatric Registry with a wide distribution of ages, ethnicities, and surgery types. Patients that underwent SG or bypass surgery had similar pre-operative characteristics, but SG patients had lower levels of ALT and AST two years after surgery, and a lower incidence of new onset abnormal ALT levels. Notably, histological and imaging studies showed that bariatric surgery, and in particular bypass surgery, are associated with an improvement in NAFLD, NASH and non-worsening of liver fibrosis [1,2,3,4,5,6,7,8,17].

Using logistic models and retrospective matching we have shown that SG is superior to RYGB and OAGB in normalizing ALT levels in both sexes. According to the logistic model, the effect of having RYGB or OAGB instead of SG on having abnormal ALT levels two years after surgery increased the odds ratio of having abnormal ALT levels by 3.05 and 5.04-fold, respectively.

Our results agree with previous registry-based studies that used one-year follow-up data [18,28]. Surgery type was not reported to affect levels of liver enzymes in a study based on the Swedish Obesity Study [6]. However, this study did not include SG patients. Studies comparing liver biopsies in NASH patients one and five-years after surgery found that RYGB was superior to gastric banding, but the study did not have enough data to compare SG to RYGB [3,8]. Several other studies that compared dozens of liver biopsies up to one year [29,30] after surgery did not find a difference between SG and RYGB, although some reported a trend toward better results following SG [30,31,32]. Our study therefore contributes to the ongoing discussion on the importance of surgery type for the treatment of obese patients by providing data from a large number of patients’ two-years after surgery, and comparing gastric bypass surgeries to SG procedure.

Improvement in liver function is associated with weight-loss and increased insulin sensitivity [33,34,35,36]. SG does not lead to greater improvements in glycemia or weight-loss than bypass surgery (this study and [13,14]). Importantly, patients who underwent bypass or SG surgery had similar demographical parameters, BMI, A1C ALT and AST levels (Supplementary Table S3). This finding suggests that surgery affects liver and plasma liver enzyme levels through both weight loss and surgery-specific mechanisms.

There is a discrepancy between our results, showing abnormal ALT levels in a relatively large fraction of patients that underwent bypass surgery, and the histological findings showing an improvement in NASH without worsening in fibrosis and improvement in histological scores after bypass surgery [3,8,17,30,31,32]. There are a few reports on the development of acute liver failure following bariatric surgery, mostly bypass surgery [2,37,38,39,40], but these cases are rare compared with the rates of abnormal ALT levels we report here.

A possible interpretation of our results is that the levels of ALT and AST after bariatric surgery, and especially bypass surgery, are not indicative of hepatic injury. If true, this finding is important for the interpretation of blood biochemistry tests of patients that had bypass surgery. Abnormal AST and ALT levels are correlated with liver injury in several hepatic conditions, including NAFLD, hepatitis, alcoholic liver disease and others, and with overall liver-related mortality [21]. However, this may not be the case after bypass surgery.

The main difference between restrictive SG surgery and malabsorptive bypass surgery is that the proximal intestine is not bypassed in SG and the pylorus is intact. The two types of surgery elicit different physiological and endocrine adaptations in the gut [12] and the resulting gut flora is different following SG and RYGB [41]. Patients who had RYGB have higher levels of bile acids in the blood than those who had SG, and the composition of the bile acids is altered [42]. How all these differences correspond to higher circulating ALT levels in some patients following bypass surgery is not clear, as there is no evidence for increased cell turnover or cellular damage after bypass surgery. However, the *ALT* gene is a target of Pparα signaling [43], and hepatic Pparα signaling is upregulated following bypass surgeries [44,45]. It is possible that the rise in ALT is a result of increased Pparα signaling and ALT expression. The causes of high ALT and AST levels particularly following bypass surgery require further research.

Our observational study has several limitations. First, it is based on levels of ALT and AST, which are crude biomarkers for hepatic injury. AST and ALT levels are only crude and do not indicate the cause, nature or even existence of a hepatic pathology [21]. A possible interpretation of the results here is that ALT levels are not indicative of liver pathology, especially following bypass surgery. Second, a follow-up period of two years is close to the nadir of weight-loss and reduction in A1C [16]. A future study with a longer follow-up is warranted. Other limitations of our study are the lack of records of patients’ medication, alcohol consumption, socioeconomic status and post-operative complications, all of which can affect obesity T2D, and levels of liver enzymes [46,47]. These data were not available in sufficient detail for most patients.

## 5. Conclusions

In this study, we compared how SG, RYGB and OAGB affect levels of liver enzymes using two-year follow-up data from thousands of patients. SG led to the greatest reduction in ALT levels, the highest rate of normalization of abnormal ALT levels, and the lowest rate of increase of ALT levels from normal to abnormal. OAGB patients experienced the greatest weight loss but were least likely to normalize ALT levels and most likely to experience a post-surgical increase in ALT levels. The large fraction of patients that display high ALT levels two years after surgery, together with the rise in the popularity of OAGB, call for further investigation into the cause of an increase in plasma ALT in a subset of patients undergoing bariatric surgery, and the diagnostic relevance of ALT and AST following bariatric surgery.

## Figures and Tables

**Table 1:** Pre- and two-year post-surgery outcomes for the three surgery types in the study population.

Parameter	SG (*n* = 4144)	RYGB (*n* = 498)	OAGB (*n* = 338)
ALT (IU/L) pre-surgery, Female	22 (17–32)	23 (16–33)	22 (16–28)
ALT (IU/L) 2 years post-surgery, Female *	14 (11–19)	19 (14–25)	21 (16.5–28)
ALT (IU/L) pre-surgery, Male	33 (25–48)	30 (22–39.75)	32 (23.5–43.5)
ALT (IU/L) 2 years post-surgery, Male *	18 (14–23)	22 (17–30.75)	24 (19.5–33)
AST (IU/L) pre-surgery	22 (18–29)	23 (18–29)	22 (19–28)
AST (IU/L) 2 years post-surgery **	18 (15–22)	22 (18–27)	24 (20–30)
Patients with abnormal ALT levels Pre-surgery	1741 (42%)	202 (41%)	133 (39%)
Patients with abnormal ALT levels 2 years post-surgery *	336 (8%)	113 (23%)	96 (28%)

ALT: alanine aminotransferase; AST: aspartate aminotransferase; SG: sleeve gastrectomy; RYGB: Roux-en Y gastric bypass; OAGB: one anastomosis gastric bypass. *p*-values derived using Kruskal-Wallis H-tests, followed by Mann-Whitney U-test for pairwise comparison for continuous variables. χ^2^ test was used for categorical variables. * *p*-value < 0.05 in a pairwise comparison of SG with RYGB and SG with OAGB; ** *p*-value < 0.05 for all surgery types pairwise compared.

**Table 2:** Pre-surgical parameters of patients with normal and abnormal ALT levels two years post-surgery.

	Normal ALT Levels Post-Surgery (*n* = 4435)	Abnormal ALT Levels Post-Surgery (*n* = 545)	*p* Value
Age (years)	44.98 (35.33–54.87)	49.53 (38.09–57.29)	<0.001
Sex—female	3029 (69%)	400 (73%)	0.01
Ethnicity (Jewish)	3705 (84%)	488 (90%)	<0.001
BMI (kg/m^2^) pre-surgery	41.14 (38.61–44.46)	40.86 (37.96–44.52)	0.1
A1C (%) pre-surgery	5.9 (5.5–6.6)	6 (5.6–6.8)	<0.001
ALT (IU/L) pre-surgery	25 (18–36)	31 (22–45)	<0.001
TG (mg/dL)	147 (108–203)	152 (110–211.7)	0.07
Surgery type (SG, RYGB, OAGB)	3797 (86%) 384 (9%) 241 (5%)	336 (61.7%) 113 (20.7%) 96 (17.6%)	<0.001
Alcohol consumption	96 (22%)	14 (26%)	0.52
Smoking	823 (19%)	108 (20%)	0.47
Hypertension	1644 (37%)	230 (42%)	0.02
OSA	740 (17%)	98 (18%)	0.44

BMI: body mass index. A1C: glycated hemoglobin. ALT: alanine aminotransferase. TG: triglycerides. SG: sleeve gastrectomy. RYGB: Roux-en-Y gastric bypass. OAGB: one anastomosis gastric bypass. OSA: obstructive sleep apnea. *p*-values derived using Mann-Whitney U-test for continuous variables and using χ^2^ test for categorical variables.

**Table 3:** Coefficients for variables in the logistic model of predicting abnormal ALT levels in matched population: over 33 IU/L for males, over 25 IU/L for females.

	β	SE β	Wald’s χ^2^	*p*-Value	OR	CI
Constant	−3.39	0.42	64.63	<1 × 10^−5^	NA	NA
RYGB vs. SG	1.11	0.26	17.78	<1 × 10^−5^	3.05	(1.82, 5.16)
OAGB vs. SG	1.69	0.25	44.01	<1 × 10^−5^	5.40	(3.28, 8.89)
OAGB vs. RYGB	0.57	0.19	8.94	<0.05	1.77	(1.22, 2.58)
Age (years)	0.02	0.01	4.98	<0.05	1.02	(1.00, 1.03)

Log-Likelihood ratio χ^2^—58.32, *p* value < 1 × 10^−5^, Wald test χ^2^—300.22, *p* value < 1 × 10^−5^. β: Coefficient, SE: Standard Error, OR: Odds Ratio, CI: Confidence Interval, NA: not applicable, SG: sleeve gastrectomy, RYGB: Roux en Y gastric bypass, OAGB: one anastomosis gastric bypass, ALT: alanine aminotransferase.

## Data Availability

The data presented in this study are available upon request from the corresponding author, due to the request of the Institutional Review Board.

## Supplementary Materials

The following are available online at https://www.mdpi.com/2077-0383/10/5/1144/s1, Table S1: characteristics of patients in the registry with two-year follow-up data on ALT levels, including only primary surgery, Table S2: Coefficients for variables in the logistic model of predicting abnormal ALT levels including the entire population study and pre-operative ALT levels, Table S3: pre- and two-year post-surgery outcomes for the three surgery types in the study population, Table S4: Retrospectively matched patients’ pre-surgical parameters for the three surgery types in the study population, Table S5: Characteristics of Responders and non-responders in reducing abnormal ALT levels 2-years after surgery divided according to surgery type.
