# Supplementary material for: Sleeve Gastrectomy Is Associated with a Greater Reduction in Plasma Liver Enzymes Than Bypass Surgeries—A Registry-Based Two-Year Follow-Up Analysis
Source: J Clin Med. 2021 Mar 9;10(5):1144. doi: 10.3390/jcm10051144 (PMC7967238; doi:10.3390/jcm10051144)
Supplement: Supplementary file 1 [file jcm-10-01144-s001.pdf]

Sleeve gastrectomy has a beneficial effect on plasma liver enzymes in comparison to bypass surgeries - a registry-based 2-year follow-up analysis

Shira Azulai[1], Ronit Grinbaum[2], Nahum Beglaibter[2], Shai Meron Eldar[3,4], Moshe Rubin[4,5], Rachel Ben-Haroush Schyr[1], Orly Romano-Zelekha[7], Danny Ben-Zvi[1*].

1. Dept. of Developmental Biology and Cancer Research, Institute for Medical Research Israel-Canada, The Hebrew University-Hadassah Medical School, Jerusalem, Israel.
2. Dept. of Surgery, Hadassah-Hebrew University Medical Center, Mount Scopus, Jerusalem, Israel.
3. Bariatric Surgery Unit, Division of General Surgery, Tel Aviv Sourasky Medical Center, Tel-Aviv, Israel.
4. Israel Sackler Faculty of Medicine, Tel-Aviv University, Tel-Aviv, Israel.
5. Department of Surgery, Tel HaShomer Medical Center, Ramat Gan, Israel.
6. Braun School of Public Health and Community Medicine, The Hebrew University of Jerusalem, Jerusalem, Israel.
7. Israel Center for Disease Control, Ministry of Health, Ramat Gan, Israel.

* Corresponding author: Danny Ben-Zvi, the Hebrew University-Hadassah Medical School, Jerusalem 91120, Israel. Tel +972-26588081, danny.ben-zvi@mail.huji.ac.il

Supporting information: Tables S1-S5

Table S1: characteristics of patients in the registry with 2-year follow-up data on ALT levels, including only primary surgery. BMI – body mass index; A1C - glycated hemoglobin; TG – triglycerides; SG – sleeve gastrectomy; RYGB – Roux en Y gastric bypass; OAGB – One Anastomosis Gastric Bypass; OSA – obstructive sleep apnea.

	Pre-surgery characteristics (n=4980)
**ALT [IU/L]**	25 [18-37]
**AST [IU/L]**	22 [18-29]
**AST/ALT**	0.89 [0.73-1.08]
**A1C [%]**	5.9 [5.5-6.6]
**TG [mg/dL]**	148 [108-204]
**BMI [kg/m²]**	41.12 [38.56-44.46]
**Age [years]**	45.39 [35.7-55.18]
**Sex - Females**	3439 (69%)
**Ethnicity**	Jewish: 4193 (84%)
**Smokers**	931 (19%)
**Alcohol consumption**	110 (7%)
**Surgery type**	SG: 4144 (83%)
	RYGB: 498 (10%)
	OAGB: 338 (7%)
**Hypertension**	1877 (38%)
**Diabetes**	1850 (37%)
**OSA**	838 (19%)

Table S2: Backwards elimination multivariate logistic model, predicting abnormal ALT levels (for men greater than 33, and women greater than 25) for all patients in the registry including ALT levels pre-surgery. β: Coefficient, SE: Standard Error, OR: Odds Ratio, CI: Confidence Interval, NA: not applicable, SG: sleeve gastrectomy. RYGB: Roux en Y gastric bypass. OAGB: one anastomosis gastric bypass. ALT: alanine aminotransferase.

predicted value-abnormal ALT levels (for men > 33, and for women > 25)

	β	SE β	Wald's χ^2	p-value	OR	CI
**Constant**	-4.51	0.26	304.42	<1e-5	NA	NA
**RYGB vs SG**	1.19	0.12	91.54	<1e-5	3.30	[2.58 4.21]
**OAGB vs SG**	1.56	0.14	130.05	<1e-5	4.75	[3.63 6.20]
**OAGB vs RYGB**	0.36	0.16	4.86	<0.05	1.44	[1.04 1.99]
**Sex - Female**	0.59	0.11	28.64	<1e-5	1.81	[1.45 2.24]
**Age [years]**	0.01	0.004	11.21	<0.01	1.01	[1.01 1.02]
**Ethnicity - Jewish**	0.53	0.16	11.46	<0.01	1.69	[1.25 2.30]

**ALT [IU/L]**	0.02	0.002	90.29	<1e-5	1.02	[1.01	1.02]

Test overall model evaluation

	χ^2	**p-value**
**Log-Likelihood ratio**	282.95	<1e-5
**Wald test**	1927.84	<1e-5

Table S3: pre- and two-year post-surgery outcomes for the three surgery types in the study population. BMI: body mass index; A1C: glycated hemoglobin; TG: triglycerides. p-values derived using Kruskal-Wallis H-tests for continuous variables and using χ^2 test for categorical variables. p-value<0.05 in post-hoc analysis [a]SG different form RYGB, [b]SG different from OAGB, and [c]RYGB different from OAGB.

	**SG (n=4144)**	**RYGB (n=498)**	**OAGB (n=338)**
**BMI [kg/m²] Pre-surgery [a]**	41.15 [38.68-44.53]	40.56 [37.85-43.80]	41.22 [38.21-44.44]
**BMI [kg/m²] 2 years post-surgery [abc]**	29.03 [26.17-32.46]	28.01 [25.19-30.82]	26.91 [24.03-29.52]
**EWL [%] [abc]**	75.02 [58.02-92.51]	79.61 [64.38-98.46]	88.81 [72.83-106.76]
**A1C [%] Pre-surgery [ac]**	5.9 [5.5-6.5]	6.2 [5.7-7.8]	6.0 [5.6-6.6]
**TG [mg/dL] Pre-surgery**	147 [107-102.03]	153 [115-214.9]	149.5 [106-2-3.5]
**Age [years] [ab]**	44.48 [34.56-54.62]	50.18 [41.38-58.6]	48.3 [37.85-57.06]
**Sex - female**	2880 (69%)	336 (67%)	223 (66%)
**Ethnicity - Jewish**	3485 (84%)	418 (84%)	290 (86%)
**smokers**	795 (19%)	79 (16%)	57 (17%)

Table S4: Retrospectively matched patients' pre-surgical parameters for the three surgery types in the study population. p-values derived using Kruskal-Wallis H-test for continuous variables and using χ^2 test for categorical variables.

	**SG (n=319)**	**RYGB (n=319)**	**OAGB (n=319)**	**p-value**
**BMI [kg/m²] pre-surgery**	41.02 [38.58-43.76]	41.04 [38.51-44.24]	41.09 [38.21-43.97]	0.94
**A1C[%] pre-surgery**	6.0 [5.6-6.55]	6.1 [5.6-6.9]	6 [5.5-6.6]	0.29
**ALT [IU/L] pre-surgery**	23 [18-32]	23 [18-32]	24 [18-31]	0.93
**TG [mg/dL]**	148 [110-203]	148 [110.5-204]	150 [106.5-205.5]	0.99
**Age [years]**	48.63 [40.25-57.91]	49.53 [40.26-58.24]	47.88 [37.89-57.82]	0.26
**Hypertension comorbidity**	142 (45%)	142 (45%)	142 (45%)	1
**Ethnicity-Jewish**	264 (83%)	264 (83%)	264 (83%)	1
**Sex-Female**	221 (69%)	221 (69%)	221 (69%)	1

Table S5: Characteristics of Responders and non-responders in reducing abnormal ALT levels 2-years after surgery divided according to surgery type. SG: sleeve gastrectomy. RYGB: Roux en Y gastric bypass. OAGB: one anastomosis gastric bypass. ALT: alanine aminotransferase

		**Responders**	**Non-Responders**
**ΔBMI [kg/m2]**	SG	12.10 [9.40-15.15]	11.20 [7.81-15.09]
	RYGB	11.81 [9.32-16.18]	11.71 [8.6-13.41]
	OAGB	13.15 [10.70-17.08]	14.66 [12.97-16.54]
**ALT pre-surgery [IU/L]**	SG	40 [33-53]	42 [33-57]
	RYGB	38.5 [31-52.5]	41.5 [35-58]
	OAGB	38 [30-49]	38 [31-62.5]
**A1C 2 years post-surgery [%]**	SG	5.5 [5.2-5.9]	5.5 [5.2-6]
	RYGB	5.6 [5.3-6]	5.8 [5.6-6.9]
	OAGB	5.5 [5.1-5.8]	5.4 [5.1-5.9]
